# Bridging the gap between pollination-related floral traits and fruit production in Asian pears

**DOI:** 10.1093/hr/uhag136

**Published:** 2026-04-13

**Authors:** Shuxuan Jing, Wufan Zhang, Yibo Luo, Jiaxing Huang

**Affiliations:** State Key Laboratory of Resource Insects, Key Laboratory for Insect-Pollinator Biology of the Ministry of Agriculture and Rural Affairs, Institute of Apicultural Research, Chinese Academy of Agricultural Sciences, Beijing 100093, China; State Key Laboratory of Resource Insects, Key Laboratory for Insect-Pollinator Biology of the Ministry of Agriculture and Rural Affairs, Institute of Apicultural Research, Chinese Academy of Agricultural Sciences, Beijing 100093, China; State Key Laboratory of Plant Diversity and Specialty Crops, Institute of Botany, Chinese Academy of Sciences, Beijing 100093, China; State Key Laboratory of Resource Insects, Key Laboratory for Insect-Pollinator Biology of the Ministry of Agriculture and Rural Affairs, Institute of Apicultural Research, Chinese Academy of Agricultural Sciences, Beijing 100093, China

## Abstract

Insect pollination is critical for fruit production, particularly for pears, most of which have high self-incompatibility and are less preferred by pollinators than other fruit trees. Research has focused on fruit traits, and less is known about how floral traits may influence pollination and fruit production. Hypothesising that pollination-related traits differ between Asian and European pears, which exhibit large variation in pollination and distinct domestication processes, we conducted a systematic review of pear pollination studies published globally from 1922 to 2025. Research on pollination has increased rapidly over the last 25 years. Compared to European pears, we found that Asian pears are more pollinator-dependent and exhibit different floral traits: lower nectar sugar concentration and lower relative content of attractive floral scents, but higher pollen production and a higher relative content of N-containing floral scents. Although Asian pear flowers attract a similar number of insect pollinator taxa as European pears, current Asian pear production still relies mainly on artificial pollination. Moreover, various artificial pollination techniques and pollination management practices, including introducing indigenous Asian honey bees, which outperformed in collecting pear pollen, had uncertain effects on pollination and fruit production, as less attention was paid to understanding the nature of plant–pollinator interactions. Further studies are needed to investigate how the domestication process and the co-adaptations of Asian honey bees influence the pollination-related floral traits of pears. This may provide valuable insights into enhancing floral attractiveness, improving pollination efficiency, and increasing fruit production in monoculture Asian pears.

## Introduction

Originating in western China during the Tertiary period, approximately 65–55 million years ago (MYA), pears have become one of the most important cultivated fruits [1]. Global pear production reached 26 million tons annually [2]. China is the leading country in pear production, accounting for 20 million tons and 78% of the world’s production, followed by the European Union, Argentina, Turkey, South Africa, and the USA [3]. Based on their geographical distribution and fruit phenotypic traits, pears can be grouped into European and Asian pears. *Pyrus communis* L. is the dominant European pear species, while Asian pears encompass various species, including *P. bretschneideri* Rehd., *P. pyrifolia* Nakai, *P. sinkiangensis* Yü, and so on. In the current pollination status of pears, European pears mostly rely on insect pollinators, whereas Asian pear production relies mainly on artificial pollination as a supplementary pollination approach [4–7]. Although there were economic motivations to increase fruit yield and quality to meet the large consumption demand, the lack of pollinators was the most important factor driving crop systems to apply hand pollination [8]. The use of chemicals in agroecosystems, the lack of semi-natural habitats for wild pollinators, and the lack of floral resources before and after the pear flowering period are the causes of inadequate pollinators for pear production of *P. sinkiangensis* in China [9]. Loss of wild and managed pollinators may have severe consequences for crop reproduction, particularly for crops with highly self-incompatible breeding systems that rely solely or heavily on cross-pollination [10, 11], such as pear.

Pears exhibit a gametophytic self-incompatibility (SI) breeding system [12–15], which requires cross-pollination with polliniser cultivars of different genotypes [5, 16–19]. As early as 1895, Waite published the first study of pear pollination, and by the 1920s, researchers began reporting that honey bees can actively transfer pear pollen for pollination [20–23]. Pollinator dependence (PD) and pollen limitation (PL) are key components in assessments of crop pollination requirements [10, 24]. PD is the amount of pollinator-associated pollination (e.g. insect and artificial pollination) required to achieve maximum pollination [24–26], reflecting the production contributed by potential pollinators [24]. PL highlights the distinction between open pollination and optimal yields, which is often achieved through supplementary hand pollination [24]. Currently, little is known about how the PD and PL differ among pear genotypes.

Pollination syndromes can be defined as suites of floral traits, e.g. flower colour, floral display size, floral scents, that include rewards associated with the attraction and utilization of particular functional groups of animal pollinators [27–33]. The concept of pollination syndrome is widely used in ecology and evolutionary biology and is regarded as a product of coevolution or co-adaptation between plants and pollinators [34]. Generally, the evolution of pollination syndrome can be considered to be driven by the differences of pollinator behaviours within and between taxonomic groups [35]. Additionally, changes at a single genetic locus may modify floral morphological traits, thereby shifting pollinator behaviours (e.g. foraging preferences and competition) and altering the availability of local pollinators [36]. Currently, there is still a lack of qualitative and quantitative understanding of pollination syndromes [34]. Although the concept of pollination syndromes is not widely used in agriculture and horticulture, several studies have observed connections between pollination-related traits and pollinator behaviours. In red clover, co-flowering diploid and tetraploid exhibit pollinator divergence: short-tongued bumble bees are more common on the shorter-corolla diploids with higher relative abundance, whereas long-tongued bees prefer the longer-corolla tetraploids [37]. The fruit production of kiwifruit [38] and blueberry [39] applied different pollination management practices, e.g. hive stocking numbers and even artificial pollination, based on knowledge of optimal pollen amount varying among cultivars.

In the case of the pear, current research has focused on fruit traits, such as texture, flavour, sweetness, and acidity [18]. Genome sequencing of 113 pear accessions from both cultivated and wild pear species revealed the two independent domestication events in Asian and European pears, and separate selective sweeps related to the different phenotypic fruit traits between Asian and European pears were also detected [15]. A recent study identified a robust selective sweep region, including genes related to fruit size, acidity, and sugar content, which provides evidence of convergent selection on fruit traits in both wild and cultivated pears [40]. However, there is not any evidence that the domestication process of pears has changed pollination-related floral traits. In fact, pear flowers were less attractive for bees compared to apple flowers in mixed fruit orchards, which was attributed to the low amount and sugar concentration in pear nectar [41–48]. The flowering of pear usually occurs before apple in the early spring, and unfavourable weather conditions may limit bee pollination [48, 49]. Recent studies have found that flowers in the Asian pear, such as *P. bretschneideri,* were less attractive to foraging honey bees than those in European pears under field observations and behavioural tests, which may be attributed to differences in floral traits [50, 51], such as floral scents of linalool [50, 51], sucrose content in nectar [51], and amino acid content in pollen [50, 51]. Nevertheless, there is a lack of knowledge on how the floral traits differed between Asian and European pear flowers and how these differences may influence the pollination process and the fruit production, so we hypothesised: (i) the pollination-related floral traits differed between Asian and European pears in attracting insect pollinators, and (ii) the diversity and foraging behaviours of insect pollinators differed between Asian and European pears.

To our knowledge, this is the first review of pear pollination at a global scale. We conducted a systematic review that specifically investigating the following aspects: (i) the historical trends in pear pollination research; (ii) the geographical distributions and pollination approaches of different pear species; (iii) PD and PL; (iv) floral traits of morphology, pollen, nectar, and floral scent; (v) diversity and foraging behaviours of pollinators; and (vi) different pollination management practices.

## Results

### Historical trends of the research topics

**Figure 1:**
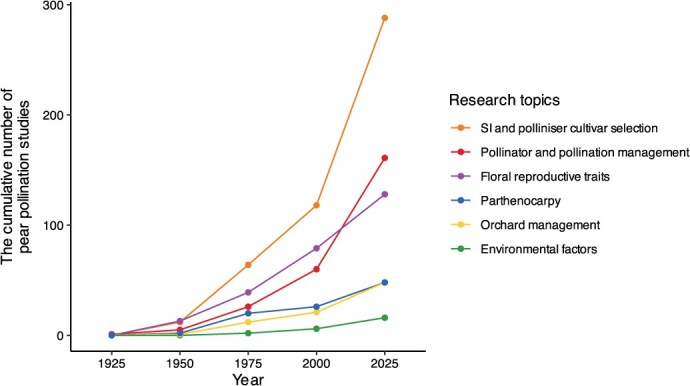
The cumulative number of pear pollination studies published from 1922 to 2025, including the following research topics: SI and polliniser cultivar selection, pollinator and pollination management, floral reproductive traits, parthenocarpy, orchard management, and environmental factors.

During the initial screening of the pear pollination studies published from 1922 to 2025, we identified 632 studies that varied in different research topics (Fig. 1). The cumulative number of studies in the SI and selection of polliniser cultivars (*N* = 288) contributed to the highest proportion (42%) of the research topics and increased rapidly over the past 25 years. The number of studies in pollinator and pollination management was the second-largest research topic (*N* = 161), increasing rapidly since 2000, encompassing various aspects of abundance and behaviour (*N* = 60), managed bee pollination (*N* = 56), artificial pollination (*N* = 42), and natural wind pollination (*N* = 3). Floral reproductive traits were the third most studied topic (*N* = 128), increasing steadily since the 1950s. The following three research topics were the least studied, and the number of studies increased slowly over the past century. Until 2025, 48 studies reported on parthenocarpy and orchard management, respectively. Orchard management practices included the use of plant growth regulator and/or plant hormone (*N* = 14), use of fertiliser, pesticides and/or fungicides (*N* = 9), flower and fruit thinning (*N* = 8), cultivar placement (*N* = 8), biocontrol including integrated pest and pollinator management (*N* = 7), and flower phenology monitoring (*N* = 2). A relatively low number of studies investigated environmental factors (*N* = 16), including temperature and climate change (*N* = 11), flora diversity, and landscape characteristics (*N* = 5).

### Geographical distributions and pollination approaches of different pear species

**Figure 2:**
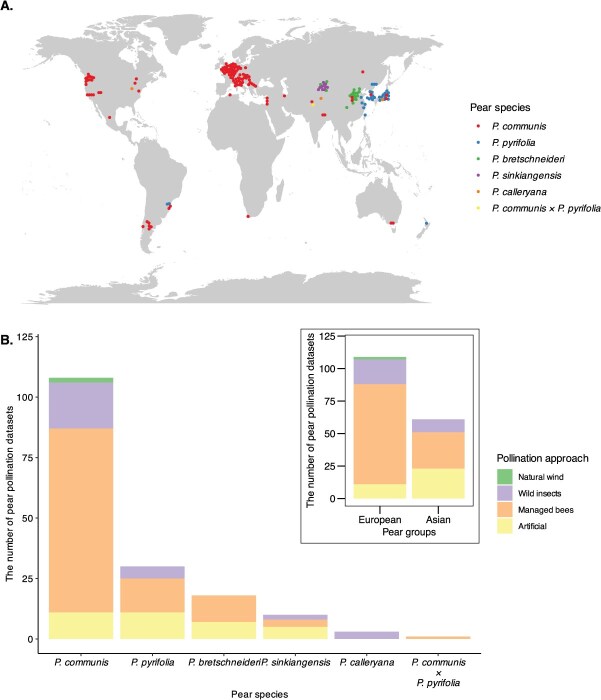
(A) The geographical distributions of pear species and (B) The proportions of studied pollination approaches categorised by pear species and pear groups (the inset in the upper-right corner) of the reviewed pear pollination 144 studies.

The geographical distributions of pear species varied in the 144 reviewed pear pollination studies (Fig. 2A). Most studies on *P. communis* were conducted in Europe, North America, and South America. Studies on *P. pyrifolia* and *P. serotina* were conducted primarily in Japan and South Korea. Studies on *P. bretschneideri* and *P. sinkiangensis* were conducted mainly in China (Fig. 2A).

In general, more pollination studies were conducted in European pears compared to Asian pears (Fig. 2B). For each pear species, *P. communis* from the European pears accounted for the most significant number of pear pollination studies (*N* = 109), which was 3.5 times of *P. pyrifolia* (*N* = 31), 6 times of *P. bretschneideri* (*N* = 18), and 11 times of *P. sinkiangensis* (*N* = 10). Among pollination approaches, most pollination studies in European pears investigated managed bees (71%) and wild insects (17%), whereas in Asian pears, studies were mainly conducted using managed bees (46%) and artificial pollination (38%). The proportion of managed bees and artificial pollination also varied among pear species. For example, managed bees were the most common pollination approach in *P. bretschneideri* (61%), whereas artificial pollination accounted for a large proportion of approaches in *P. sinkiangensis* (50%). The number of studies investigating wild insects on Asian pears is relatively low, with five on *P. pyrifolia*, three on *P. calleryana*, two on *P. sinkiangensis*, and none on *P. bretschneideri*. Two studies in *P. communis* investigated the potential of natural wind for pear pollination.

### Comparison of pollinator dependence and pollen limitation between Asian and European pear groups

**Figure 3:**
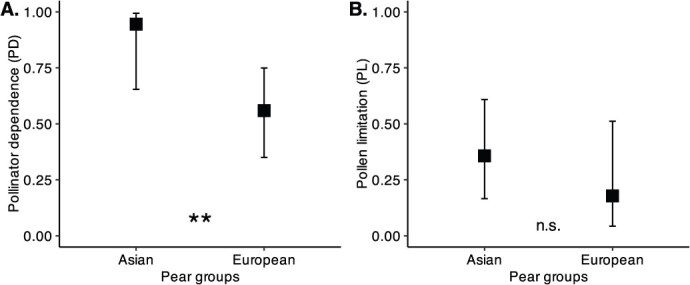
Comparison of (A) PD between Asian (*N* = 5) and European (*N* = 7) pear groups; (B) PL between Asian (*N* = 6) and European (*N* = 4) pear groups. Error bars indicate the 95% CI of the EMMs. ^**^P = 0.0017, n.s. = non-significance (*P* > 0.05).

For PD, there was a significant effect of pear group on PD values [generalized linear model (GLM), likelihood ratio test (LRT) = 9.84, degree of freedom (df) = 1, *P* = 0.0017, Fig. 3A]. The PD value in Asian pear was 0.95 with 95% CI (0.65, 0.99), which was significantly higher than that of European pear, which had a PD value of 0.56 with 95% CI (0.35, 0.75). Within the Asian pear group, we compared the PD values between the insect and artificial pollination and found that there was no significant effect of pollination approach (GLM, LRT = 0.06, df = 1, *P* = .8084, Fig. S1).

For PL, there was a non-significant effect of pear groups on the PL values (GLM, LRT = 1.35, df = 1, *P* = .2454, Fig. 3B). The PL value in Asian pear was 0.36 with 95% CI (0.17, 0.61), which was slightly higher, but non-significant compared to that of European pear that had PD value of 0.18 with 95% CI (0.043, 0.51).

### The diversity of pollination-related floral traits between the Asian and European pear groups

Five studies investigated the number of flowers per inflorescence (Table S5), and six studies examined the flower diameter (Table S5). There were no significant differences in the number of flowers per inflorescence [Linear model (LM), *F* = 0.53, df = 1, *P* = .4801, Fig. S2A] and the flower diameter (LM, *F* = 0.04, df = 1, *P* = .8401, Fig. S2B) between Asian and European pear flowers. The mean number of flowers per inflorescence in Asian pear was 7.52 with 95% CI (6.24, 9.06) and in European pear was 6.81 with 95% CI (5.07, 9.14). The mean flower diameter in Asian pear flower was 3.33 cm with 95% CI (2.86, 3.79) and in European pear flower was 3.42 cm with 95% CI (2.37, 4.46).

**Figure 4:**
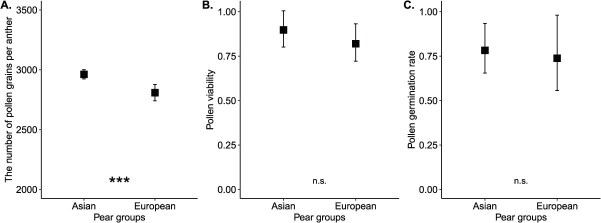
Comparison of pollen traits between Asian and European pear groups: (A) the number of pollen grains per anther; (B) pollen viability; (C) pollen germination rate. Error bars indicate the 95% CI of the EMMs. ^***^P < 0.001, n.s. = non-significance (*P* > 0.05).

There were ten studies investigated pollen traits (Table S6), and 11 studies investigated nectar traits (Table S7). For the quantity and quality of pollen, the number of pollen grains per anther in Asian pear was found to be significantly higher than that of European pear (GLM, LRT = 18.65, df = 1, *P* < 0.001, Fig. 4A). Both pear groups reached more than 2500 pollen grains per anther (Fig. 4A). There was non-significant difference between Asian and European pears in pollen viability (GLM, LRT = 1.32, df = 1, *P* = .2503, Fig. 4B) and pollen germination rate (GLM, LRT = 0.16, df = 1, *P* = .6914, Fig. 4C). Both pollen viability and pollen germination rate were relatively high in all investigated pear groups, which were higher than 0.75. For the quantity and quality of nectar, there was no significant difference between Asian and European pears in nectar volume per flower (LM, *F* = 0.02, df = 1, *P* = .8955, Fig. S3A). The average nectar volume per flower in Asian pears was 2.57 μl with 95% CI (0.98, 6.70), and in European pears it was 2.71 μl with 95% CI (0.84, 8.76). The sugar concentration seemed higher in European pear flowers (on average 20%) compared to Asian pear flowers (on average 8%), but was non-significant (GLM, LRT = 1.51, df = 1, *P* = .2184, Fig. S3B).

**Figure 5:**
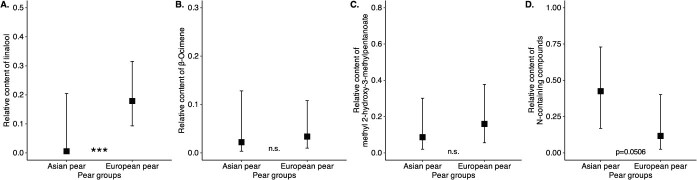
Comparison of the relative content in volatile organic compounds between Asian and European pear flowers: (A) linalool; (B) β-ocimene; (C) methyl 2-hydroxy-3-methylpentanoate; (D) N-containing compounds. Error bars indicate the 95% CI of the EMMs. ^***^P < 0.001, n.s. = non-significance (*P* > 0.05).

Five studies quantitatively investigated the floral scents of pear (Table S8). There was a significant difference of relative content of linalool between Asian and European pear flowers (GLM, LRT = 13.41, df = 1, *P* <0.001, Fig. 5A). The relative content of linalool in European pear flowers was 17.8% with 95% CI (9.3%, 31.5%), which was significantly higher than that of Asian pear flowers, 0.58% with 95% CI (0.01%, 20.1%). The relative content of N-containing compounds in Asian pear flowers was slightly higher than that of European pear flowers (GLM, LRT = 3.82, df = 1, *P* = 0.0506, Fig. 5D). There was no significant difference between Asian and European pear flowers in the relative content of β-ocimene (GLM, LRT = 0.19, df = 1, *P* = .6602, Fig. 5B) and methyl 2-hydroxy-3-methylpentanoate (GLM, LRT = 0.69, df = 1, *P* = .4058, Fig. 5C).

### Diversity and foraging behaviours of insect pollinators

**Figure 6:**
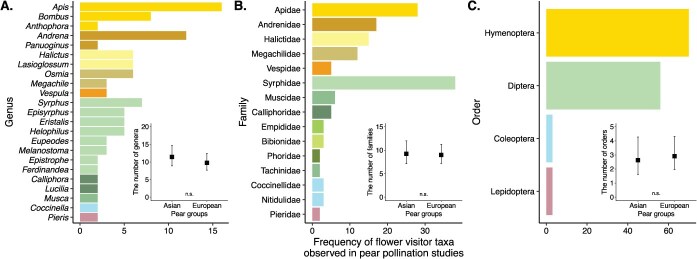
Frequency of insects observed from pear pollination studies (*N* = 19) in the taxonomic category of: (A) genus; (B) family; (C) order. The insets in the lower-right corner within each taxonomic category showed no significant difference between Asian and European pear groups in the number of genera, families, and orders. Error bars indicate the 95% CI of the EMMs. n.s. = non-significance (*P* > 0.05).

In total, we included 19 studies reporting the diversity of insect pollinators across four orders, 15 families, and 23 genera, each occurring at least twice in the investigated studies. Seven out of 19 studies reported the flower visitors at the species level (Table S9), among which 80 species were reported from five studies in *P. communis*, 19 species were reported in one study in *P. sinkiangensis*, and 18 species were reported in one study in *P. calleryana*. Insect pollinators, including wild insects and managed bees, contributed largely to all studied pear species, 88% of the studies in European pears and 62% of the studies in Asian pears relied on insect pollinators (Fig. 2B). Most of the observed flower visitors came from Hymenoptera, including 5 families and 10 genera (Fig. 6). Dipteran insects contributed to the second abundant order, including 7 families and 11 genera. Syrphidae accounted for the most observed flower visitors at the family level, followed by Apidae, Andrenidae, Halictidae, and Megachilidae. *Apis*, *Andrena*, and *Bombus* contributed to most of the observed pear flower visitors for the genus category. Comparing the number of taxa, the differences between Asian and European pears were non-significant at different levels of genus (GLM, LRT = 1.02, df = 1, *P* = 0.3134, Fig. 6A), family (GLM, LRT = 0.031, df = 1, *P* = .8586, Fig. 6B), and order (GLM, LRT = 0.13, df = 1, *P* = .7135, Fig. 6C).

**Figure 7:**
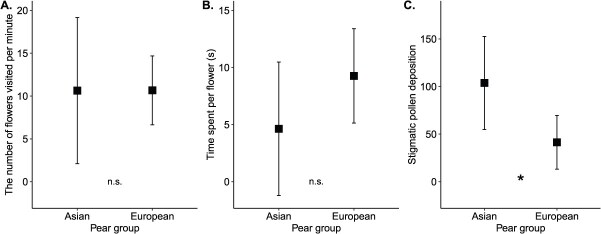
Comparison of different foraging behaviours between Asian and European pear pollination studies: (A) the number of visited flowers per minute; (B) time spent per flower; (C) stigmatic pollen deposition. Error bars indicate the 95% CI of the EMMs. ^*^P = 0.0172, n.s. = non-significance (*P* > 0.05).

We investigated 12 studies that measured specific foraging behaviours: the number of visited flowers per minute (*N* = 6), time spent per flower (*N* = 5), and stigmatic pollen deposition (*N* = 4), based on a single visit of insect pollinators (Fig. 7 and Table S10). Among the studied pear species, *P. communis* was the most investigated pear species (*N* = 8), followed by *P. pyrifolia* (*N* = 3). There was only one study on *P. bretschneideri*, and none on *P. sinkiangensis*. There was a non-significant effect of pear groups on the number of flowers visited per minute (LM, *F* = 0.0001, df = 1, *P* = .9928, Fig. 7A) and the time spent per flower (LM, *F* = 3.36, df = 1, *P* = .1095, Fig. 7B). For the stigmatic pollen deposition, there was a significant effect of pear groups (LM, *F* = 10.66, df = 1, *P* = 0.0172, Fig. 7C). The number of pollen grains deposited on stigma in Asian pear was on average 103.70 with 95% CI (54.80, 152.70), which was significantly higher than that of European pear, on average 41.40 with 95% CI (13.20, 69.70).

### Pollination management practices to improve pear pollination

Different management practices applied to improve pollinator behaviour and fruit yield were summarised from 10 studies (Table S1). The most common practices were using bee attractants (*N* = 5 studies), manipulating floral rewards (*N* = 2 studies), and bee hive management (*N* = 2 studies). The most improved pollination behaviours were abundance, pollen collection, and visitation rate. However, only a few studies measured the fruit production in terms of fruit set, fruit size, and fruit yield. *Apis mellifera* was the studied pollinator species in all studies, and 80% of the studies investigated these management practices in European pear of *P. communis*, there was one study that investigated Asian pears of *P. bretschneideri* and *P. sinkiangensis*, respectively.

**Figure 8:**
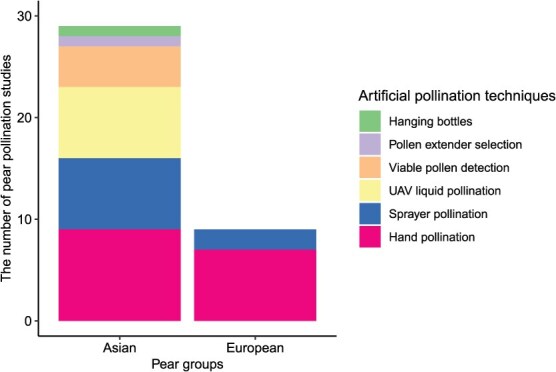
The number of pear pollination studies investigating different techniques in relation to artificial pollination approaches in pear groups of Asian and European pears.

Thirty-three studies investigated detailed aspects of artificial pollination approaches in Asian and European pear groups (Fig. 8 and Table S11). Overall, the number of studies on artificial techniques in Asian pears (*N* = 24) was 2.6 times that of the European pears (*N* = 9). Hand pollination and sprayer pollination were the most commonly studied methods in both groups. The artificial pollination techniques also varied across Asian pear species (Fig. S6). For example, liquid pollination using unmanned aerial vehicle (UAV) was studied mainly in *P. bretschneideri* and *P. sinkiangensis*, and viable pollen detection was studied primarily on *P. pyrifolia* and *P. bretschneideri* (Fig. S6).

## Discussion

### Asian pear monocultures are highly pollinator-dependent for fruit production

Historically, research on pollination ecology has increased significantly since the 1990s [52]. We also found that the number of studies on pollinator and pollination management in pear pollination increased rapidly over the past 25 years. However, the number of which was only half of the breeding studies (Fig. 1). Geographically, the differences of distributions in studied pear species and research topics of pear pollination studies between Asian and European pears were large (Fig. 2A). The number of pear pollination studies conducted in European pears was twice the sum of studies in Asian pear species (Fig. 2B**)**. This asymmetrical research interests contrast with the current global pear production, where Asian countries are leading the pear fruit production, but less research is conducted in relation to pear pollination.

At the global scale, the rapid expansion of monoculture crops highly dependent on pollinators has faced challenges, including a lack of agricultural diversity, which may hamper the diversity of pollinator communities and crop production [53]. This is also true for large monocultures in Asian pear production. We found that both Asian and European pears are highly pollinator-dependent and pollen-limited (Fig. 3), similar to many fruits, such as blueberries and apples [54]. According to previous studies evaluating the PD values in crops, the PD value of 0.56 for European pears in the current research can be classified as ‘high’, which agrees with a previous study [24] that evaluated the PD values in European pears. Compared to European pears, Asian pear groups even obtained significantly higher PD value of 0.95 (Fig. 3A), which can be classified as ‘essential’ [10, 24], showing that the current large monocultures of Asian pears are more pollinator-dependent for fruit production.

For pollinator-dependent crops, PD values reflect the distance between auto-pollination and optimal pollination [24], which is calculated based on the crop’s breeding system. The high PD values in Asian pear groups might be related to the lower ability for parthenocarpy in many genotypes of Asian pears, e.g. *P. pyrifolia* and *P. bretschneideri* [55, 56]. They might also be related to high SI [5, 19, 57]. Only a few genotypes in Asian pears are self-compatible, such as *P. bretschneideri* [58, 59] and *P. pyrifolia* [60]. The low level of parthenocarpy, combined with high SI, may pose challenges for Asian pear production, including selecting optimal polliniser cultivars and designing pear orchards.

In the current study, we used fruit set as an index of pear production to calculate PD values. Nevertheless, the fruit set may not fully reflect the plant fruit production, as some pear species may have a high number of flowers and a low fruit set but produce heavy fruits and eventually yield a high weight [22]. Currently, more than 3000 pear varieties are grown globally [61]. Yet, there is a notable lack of comparisons of PD values at the variety and/or cultivar level [24]. In the future, more comparisons will be needed to fully understand the contributions of each pollination approach to fruit yield, including fruit set, fruit weight, and seed set.

### Distinct pollination-related floral traits between Asian and European pears

As higher PD values were found in Asian pears compared to European pears, we also expected that Asian pears might be more pollen limited. Surprisingly, although PL values appeared higher in Asian pears compared to European pears, a non-significant difference was found (*P* = .2454, Fig. 3B). PL values reflect the distances between open pollination and optimal pollination [24], and the calculation of which was based on the pollination services received by the crop under open pollination conditions. Upon examining the details of the included studies used to calculate PD and PL values in the current study, we found that studies reporting pollinator-associated production primarily attributed PD values to the managed bee spec*ie*s, *A. mellifera* (Table S2). In calculating PL values, open pollination was contributed by both wild and managed bees in Asian and European pears (Table S3). In pear orchards, it is impossible to exclude the effects of supplementary managed bees in contributing to pear pollination. Given similar PL values, we suggest that effective pollination services for Asian pears should be expected when wild and managed bees are available, just as they are for European pears.

Hymenopteran and dipteran insects are the largest functional groups of insect pollinators in pear pollination, contributing to 95% of the pear flower visitors in previous studies (Fig. 6C). In our second hypothesis, we expected that there would be differences in the diversity and foraging behaviours of insect pollinators between Asian and European pears. Only seven studies reported pollinator taxa at the species level (Table S9), with only two studies on Asian pears. The lack of studies may affect the comparison of distinct pollinator communities between Asian and European pear flowers. With the limited data, we found that the number of insect pollinator taxa was similar at the taxonomic categories of order, family, and genus (Fig. 6), indicating that, regardless of abundance, the taxonomic richness of insect pollinators was similar between Asian and European pears. Asian pear flowers may attract as many insect pollinator groups or species as European pear flowers, which agrees with our findings on PL values, showing that pollination services in the studies were provided by both wild and managed bees under open pollination conditions (Fig. 3B and Table S3).

As the distinct phenotypic traits in fruit were involved in the domestication processes of Asian and European pears [15], we expected distinct pollination-related floral traits to have been selected during the domestications of Asian and European pears, as outlined in our first hypothesis. Partly in agreement with our hypothesis, we found non-significant differences between Asian and European pear flowers in the number of flowers per inflorescence (*P* = .4801, Fig. S2A) and in flower diameter (*P* = .8401, Fig. S2B). For other morphological traits, the colour of anthers and petals, as well as the number of stamens and pistils, were measured in a few studies of Asian pear species of *P. betulifolia* [46] and *P. pyrifolia* [62]. It was suggested that petal colour does not explain the differences in visitation rates of pollinators by comparing the chromatic contrast of different cultivars in Asian pear (*P. pyrifolia)* [63].

In terms of floral rewards, the number of pollen grains per anther was significantly higher in Asian pears compared to European pears (Fig. 4A), but not in pollen viability (Fig. 4B) and germination rate (Fig. 4C). High-quality pollen delivered by pollinators enhances fruit set and seed yield by promoting pollen germination and pollen tube growth [64]. A previous study compared the pollen quality and SI between Asian and European pears and concluded that most Asian pears had high pollen yields, viability, and germination rate [57]. We also found significantly higher stigmatic pollen deposition by hymenopteran pollinators in Asian pear flowers compared to European pear flowers (Fig. 7), which might be related to the varying capacities of stigmas to receive pollen, which is yet to be determined, or to the substantial pollen supplied by Asian pears (Fig. 4A). We found lower sugar concentration in Asian pear flowers (on average 8%) compared to European pear flowers (on average 20%, Fig. S3B), and insect pollinators seemed to spend less time per flower in Asian pear flowers compared to European pear flowers (Fig. 7B). Therefore, despite the high pollen production, the low quality of nectar may limit the floral attractiveness of Asian pear flowers.

To attract insect pollinators with floral rewards and facilitate pollination, floral scents are essential for communication between insects and flowering plants [65]. The relative content of linalool in Asian pear flowers was found to be significantly lower than that of European pear flowers (Fig. 5A). Linalool was one of the most abundant compounds in European pears [66]. Our results suggest that a lack of linalool may be one reason for the lower floral attractiveness of Asian pear flowers compared to European pear flowers. The relative content of another monoterpenoid, β-ocimene, was similar between Asian and European pear flowers (Fig. 5B). Methyl 2-hydroxy-3-methylpentanoate, a floral volatile compound found abundant in European pear flowers [66], also showed a similar relative content in Asian pear flowers (Fig. 5C). Interestingly, Asian pear flowers had higher relative content of N-containing compounds (*P* = 0.0506, Fig. 5D), which might be related to the higher pollen production in Asian pear flowers (Fig. 4A). The floral volatile compounds and the pollen odours can be discriminated by pollen-feeding insects, including honey bees [67, 68]. Although the scents from these N-containing compounds may be ‘unpleasant’ for human noses, these compounds may have played important roles in attracting pollinators to visit pear flowers, for bees preferred to collect pollen from pear flowers. Moreover, the limonene, a monoterpene that was not preferred by honey bees, was found to be abundant in three cultivars of *P. bretschneideri,* with relative contents of 15.96%–41.71% [69], providing evidence that Asian pear flowers may also emit floral volatile compounds that decrease the pear flower attractiveness.

While the current European pear production relies mainly on insect pollination, Asian pear production mostly relies on artificial pollination, which may be attributed to the limited number of pollinators in large Asian pear monocultures [9]. From the perspective of pollination-related floral traits, there are obvious differences between the flowers of Asian pear and European pear. The relatively low sugar concentration in nectar and the lack of attractive floral volatile compounds may limit the attractiveness of Asian pear flowers. On the contrary, Asian pear flowers have high pollen production, which responds well to the behaviour of Asian honey bees, dedicating themselves to collecting pollen from Asian pear flowers [70]. At present, it is unclear whether these pollination-related floral traits of Asian pear flowers resulted from co-adaptation between wild Asian pear and Asian honey bees or from long-term domestication of Asian pear. However, it is certainly true that these pollination-related traits of Asian pear have contributed to the current limitation of pollination services for fruit production.

### Is artificial pollination the solution for Asian pear production?

The current efforts to improve Asian pear pollination have focused on developing artificial pollination techniques (Fig. 8). Despite the availability and widespread use of various artificial pollination techniques (Fig. 8), it remains unclear whether the artificial pollination approach is more efficient for improving Asian pear production. We also compared PD values between insect and artificial pollination approaches in Asian pears. The results showed non-significant difference between the two pollination approaches (*P* = .8040, Fig. S1). Nevertheless, evaluating the effect of each artificial pollination technique is challenging due to significant variations in local environmental conditions and application methods. For example, the efficiency of UAV pollination is influenced by spray volume per unit area, flight altitude, and the flight speed [71]. Economically speaking, artificial pollination is labour-intensive and time-consuming [7, 72–74]. More than half of the pollen used in artificial pollination in Japan was imported [75]. One kilogram of pear pollen grains requires 150 kg of fresh pear flowers [76]. It was reported that artificial pollination accounted for approximately 40% of the annual working time in pear production [5, 77].

Compared with artificial pollination, less attention has been given to pollination management practices in Asian pears, with most studies focusing on European pears (Table S1). The effects of current pollination management practices on pollinator behaviour and fruit yield remain uncertain, as reviewed previously [78]. In European pears, feeding syrup scented with pear mimic odour may establish olfactory memories, improving pollen collection and yield [79]. Sequentially introducing honey bee hives (1.25 colonies per hectare) at 10% flowering and full flowering in pears may increase the number of bees per tree and eventually improve fruit yield, due to the floral rewards supplied by the mass flowering of pears [49]. In Asian pears, honey bee pollen collection behaviour can be enhanced through pollen trapping and syrup feeding [72]. Currently, only a few management practices utilised knowledge of pollination-related floral traits and floral resources. Future management practices could be improved by integrating floral attractiveness with floral rewards.

Moreover, the ecological traits of crops and their interactions with microbes and pollinators may affect the sustainability of agroecosystems [80]. Nectar-inhabiting organisms may influence the volatile compounds of nectar and, in turn, affect the floral attractiveness to pollinators [81]. In the case of pear, inoculation of yeast *Metschnikowia reukaufii* and bacterium *Acinetobacter nectaris* in *P. communis* increased the visitation rate of honey bees and hoverflies. Still, it did not increase fruit or seed set [82]. Further studies are needed to integrate pollination management with floral resources and plant–pollinator–microorganism interactions to improve pear production, particularly in Asian pears.

### Future perspectives

The divergence between Asian and European pears was estimated to have occurred between 6.6 and 3.3 MYA, and animals may have mediated this divergence through fruit and pollen dispersal [15]. The domestication of Chinese pear species began 3000 years ago, and commercial pear orchards have existed for more than 2000 years [1]. Among hymenopteran insects, Asian honey bees (*Apis cerana* Fabr.) have been utilised for pollination and honey production across Asia for thousands of years [70, 83]. Compared to *A. mellifera*, workers of *A. cerana* forage on dispersed floral resources [84]. Before the start of modern pear breeding in China in 1956, which marked the beginning of large-scale monoculture of Asian pears [85], the small, scattered cultivation of Asian pears matched well with the foraging behaviour of Asian honey bees. However, the current large monocultures of Asian pears have apparently mismatched the dispersed floral resources foraging habits of *A. cerana* workers. This mismatch between monoculture Asian pears and Asian honey bees has been suggested to prevent the bees from providing adequate pollination services for current Asian pear production, which is heavily dependent on artificial pollination.

Since modern pear breeding began, the genetic basis and molecular regulatory mechanisms underlying fruit quality traits in pear have been studied intensively [85, 86]. Fruit quality traits, such as colour, aroma, flavour, and texture, as well as nutritional traits, play essential roles in shaping fruit flavours and consumers’ choices in the long-term domestication and breeding history of pear [87]. Connecting the biosynthesis of volatile compounds between flowers and fruits may provide insights into how breeding activities may improve fruit aromas and modify floral volatile compounds. For example, the signalling of plant phytohormones jasmonate play important roles in regulating flower development, attractiveness for pollinators, and floral defence functions [88], and methyl jasmonate may influence the fruit aroma by changing the relative content of the flower volatile compound phenylethyl alcohol, namely, phenethyl alcohol [87], a compound which was also found in Asian pear flowers [69]. Moreover, in olives and some tropical tree species, a close relationship between the maximum pollen emission during flowering and fruit production was found [89, 90].

However, during the 3000 years of domestication of Chinese pear species [1], pollination-related floral traits have been relatively neglected. Even in modern science eras, although there was a large number of studies investigating floral reproductive traits in the research topics (Fig. 1), very few studies connected the pollination-related floral traits to the foraging behaviours and the pollination contributions of different insect pollinators (but see studies of European [44, 45, 48] and Asian pears [62]). The most important point is the lack of information on changes in these traits during the long history of domestication of the Asian pear. Further understanding of changes in pollination-related floral traits is urgently needed. With comprehensive knowledge of pollination-related floral traits of Asian pears, it is possible to carry out intensive breeding activities to balance floral attractiveness, pollination, fruit yield, and favoured fruit quality traits.

Generally, there are two strategies to improve pollination in Asian pear production. One is based on the ecological relationships between plants and pollinators to improve pear floral attractiveness and floral rewards, e.g. by moderating the relative abundance of attractive and unattractive volatile compounds or by altering nectar sugar concentrations, which requires integrating plant breeding activities with pollination biology research. From the pollinator perspective, it is possible to breed indigenous honey bees better suited to provide pollination services in monocultures of Asian pears. The interaction between pear flowers with diverse wild pollinators is yet to be determined. A recent study used bags with different mesh sizes to determine the pollination contributions of the families, such as Andrenidae, Halictidae, and Syrphidae in Asian pear orchards [19]. To date, only a few studies have compared pollination-related floral traits between wild and cultivated pear populations [91, 92]. More studies are needed to link pollination-related floral traits to the foraging behaviours of different insect pollinators in both wild and cultivated pear populations, which may provide insights into the influence of domestication on pollination syndromes.

Another strategy is based on genetic improvement. Breeding activities in terms of SI and the selection of polliniser cultivars are leading the research topics since 1950s and contributing to 42% of the total number of pear pollination studies (Fig. 1). Currently, increasing the self-compatibility are becoming one of the primary breeding objectives in fruit trees to improve the genetic variability and enlarge the selection range of polliniser cultivars, as high SI may limit the cross-pollination and the following fruit set and fruit production, especially for monocultures [93]. For the selection of polliniser cultivars, only three studies were conducted on European pears [94–96]. This reveals an empirical gap in practical orchard management with pollinators. Therefore, for Asian pears, which are more dependent on pollinators than European pears, it is crucial to improve Asian pear pollination by properly selecting polliniser cultivars, designing orchards, and enhancing cross-pollination with insect pollinators that deliver sufficient, viable, and compatible pollen [97].

## Conclusions

We conducted a comprehensive systematic review comparing Asian and European pears to present an overview of pollination in global pear production. We found that both Asian and European pears are highly pollinator-dependent and pollen-limited. However, unlike European pear production, which relies primarily on wild and managed insect pollinators, current Asian pear production applies artificial pollination extensively to increase fruit yield. Compared with European pears, Asian pears have higher pollen production and a higher content of N-containing floral volatile compounds, providing sufficient pollen for pollen collectors, but lower nectar sugar concentration and a lower relative content of attractive floral volatile compounds may limit floral attractiveness in Asian pears. The domestication process and breeding goals for improving fruit yield and fruit quality traits may change pollination-related floral traits, leading to a lack of co-adaptation between insect pollinators and Asian pears. Due to the high cost of artificial pollination and the varied effects of pollination management practices, further research is needed to investigate how to increase the floral attractiveness of Asian pear flowers, to improve the co-adaptations between indigenous honey bees and Asian pear flowers, and to adjust the pollination management practices to be more effective for attracting insect pollinators, increasing pollination efficiency, and improving pear fruit production.

## Materials and methods

### Data collection and information extraction

A systematic review was performed by searching the literature in Web of Science with the following search terms: ‘pear’ AND ‘pyrus’ AND ‘pollinat*’. We conducted an initial search that yielded 1455 research articles and proceedings papers (accessed 12 August 2025). The included databases are shown in Fig. S4. We screened the studies by reading their titles and abstracts. As we focused on pear pollination, we excluded studies that examined only breeding activities, e.g. reports of new pear cultivars. We also excluded studies that used the search terms ‘pear’ and ‘pollination’ as examples in the text, as well as duplicates and reviews.

For the different research topics, we sorted these studies during the initial screening: (i) SI and polliniser cultivar selection; (ii) pollinator and pollination management; (iii) floral reproductive traits; (iv) parthenocarpy; (v) orchard management; and (vi) environmental factors. In total, we yielded 632 studies.

To examine the geographical distributions and pollination strategies of the studied pear species, we reviewed the full texts of studies on pollinators and pollination management. The registered pear species in the current study were European common pear (*P. communis*), Japanese pear (*P. pyrifolia*), Chinese white pear (*P. bretschneideri*), Korla fragrant pear (*P. sinkiangensis*), Callery pear (*P. calleryana* Decne.), and an interspecific hybrid of semisoft pear (*P. communis* × *P. pyrifolia*). As synonyms of *P. pyrifolia*, studies conducted in *P. serotina* Rehd. were registered in the *P. pyrifolia* group [98]. We further divided the pear species into two groups: European pears, including *P. communis*, and Asian pears, including the remaining pear species mentioned above, based on their geographical distributions [99, 100] and genetic variation [15, 40].

For the pollination approaches, we sorted the studies with the following aspects: (i) artificial pollination, including hand pollination, hanging bottles with polliniser flowering branches, sprayer pollination (using hand or electrostatic sprayer), UAV liquid pollination, *etc*.; (ii) managed bees, e.g. using managed bee hives of honey bees and bumble bees; (iii) wild insects, which were observed under field conditions; and (iv) natural wind pollination, pollination that rely on natural ventilation. In total, we investigated 144 studies.

To compare PD and PL values between Asian and European pear groups, we extracted data from 144 studies that investigated the pollination process in detail. In total, we identified 12 studies on PD values (Table S2) and 10 on PL values (Table S3), including four additional studies from the reference list. For both Asian and European pears, we followed the calculation of PD value [24], using the following equation:


\begin{align*}& \mathrm{Pollinator}\ \mathrm{dependence}\ \left(\mathrm{PD}\right)\\&\quad=1-\frac{\mathrm{Pollinator}\ \mathrm{exclusion}\ \mathrm{production}}{\ \mathrm{Pollinator}-\mathrm{associated}\ \mathrm{production}} \end{align*}

In the calculation, we used fruit set to represent pollinator production. When information on fruit set was unavailable, we used other reproductive traits related to fruit quality and/or seed set. If a study measured reproductive traits over more than 1 year and/or evaluated several cultivars, we calculated the mean PD and obtained a single value from each study. Pollinator-exclusion production refers to fruit set occurring without pollinators, and pollinator-associated production refers to fruit set achieved through insect and artificial pollination [24]. In comparing PD between Asian and European pear groups, pollinator-associated production refers to the pollination contributed by insect pollinators, including both wild insects and managed bees, as only a few studies differentiated the pollination contributions of these groups. We further compared PD values between the insect and artificial pollination approaches for the Asian pear group, whose fruit production relied heavily on artificial pollination (Table S4). PD values can be classified into four degrees: little (0–0.09), modest (0.10–0.39), high (0.40–0.89), and essential (0.90–1.00) [10].

For both Asian and European pears, we followed the previous calculation of PL value [25, 26], using the following equation:


\begin{align*}& \mathrm{Pollen}\ \mathrm{limitation}\ \left(\mathrm{PL}\right)\\&\quad=1-\frac{\mathrm{Open}\ \mathrm{pollination}\ \mathrm{production}}{\ \mathrm{Supplementary}\ \mathrm{hand}\ \mathrm{pollination}\ \mathrm{production}} \end{align*}

In the PL value calculation, we used fruit set as the main reproductive trait, as in the PD value calculation. Open pollination production was provided by wild insects and managed bees present in the pear orchards. We selected artificial pollination studies that used compatible cross-pollen to avoid underestimating the effects of artificial pollination [26].

To understand the pollination-related floral traits of different pears, we investigated the studies on floral reproductive traits. As the initial search may not include all the information related to the floral traits, we conducted a second search in WOS with the following terms: ‘pear’ AND ‘pyrus’ AND ‘flower characteristics’, and ‘pear’ AND ‘pyrus’ AND ‘floral traits’, which yielded 493 research articles (accessed 17 September 2025), and 14 studies were included for further investigation based on the information available. We specifically investigated the floral traits that directly influence pollinator behaviour. Flower morphology included the number of flowers per inflorescence and flower diameter. Pollen traits included quantitative traits of the number of pollen grains per anther and qualitative traits of pollen viability (using staining method with dyes) and pollen germination rate (*in vitro* germination test in culture media). There are various methods available for testing pollen quality [101]. In practice, pollen viability usually shows higher fertility than *in vitro* germination, leading to a systematic overestimation of pollen quality [102]. Therefore, we included both methods in the current study that are widely used in pear pollination studies. Because many studies have measured pollen quality for artificial pollination, this current analysis included only data on floral traits collected under open-field (i.e. *in situ)* conditions. Nectar traits included nectar volume per flower and sugar concentration. In terms of the floral scents, we investigated terpenoids, including linalool and β-ocimene, as well as other abundant volatile compounds, methyl 2-hydroxy-3-methylpentanoate, and N-containing compounds. Terpenoids, such as linalool and β-ocimene, play crucial roles in attracting insect pollinators [66, 103], and linalool was found to be one of the most abundant compounds in European pears [66]. Methyl 2-hydroxy-3-methylpentanoate is one of the floral volatile compounds that were rarely detected in other floral scents, but were found in pear flowers [66]. Some pear flowers (e.g. *P. calleryana* and *P. kawakamii* Hayata) emit unpleasant scents to human noses, which are attributed to nitrogenous amines in floral volatile compounds [104]. It was suggested that the mixture of various N-containing compounds is responsible for the unpleasant scents in European pear flowers [66].

During the investigation, more pear species were identified. We grouped them into either Asian, e.g. *P. betulifolia* Bunge, *P. ussuriensis* Maxim, or European pear groups, e.g. *P. caucasica* Fed., *P. georgica* Kuth., *P. vsevolodii* Heideman., and *P. salicifolia* Pall., according to the previously studied genetic groups [105, 106].

To examine the diversity of insect flower visitors, we extracted information from 19 studies that at least reported to the order level. Due to the large number of insect species reported, we sorted the taxonomic units of the flower visitors at the three levels: (i) genus, (ii) family, (iii) and order [107]. The family level was collected whenever available for those who did not report at the genus level. Each taxonomic level was presented when the frequency of flower visitors was reported at least twice in the included studies. We also registered the insect species names for those studies that reported and supplied them in the supplementary materials. We included only studies conducted under field conditions. For foraging behaviours, we focused on the Hymenoptera functional group, following the previous classification of pollinator functional groups [28, 108]. We extracted the data from that reported the foraging behaviours of: (i) the number of visited flowers per minute; (ii) time spent per flower; and (iii) stigmatic pollen deposition. When studying several insect species, we included the foraging behaviour of each species as a single dataset. In total, we yielded 28 datasets from 12 studies.

Lastly, we investigated the pollination management practices and artificial pollination techniques. From the 144 studies that studied pollinator and pollination management, we yielded ten studies covering the following management practices: (i) using bee attractant (e.g. synthetic Nasonov gland pheromone, queen mandibular pheromone, and bee scent); (ii) manipulating floral resources (e.g. pollen trapping and scented sucrose feeding); (iii) bee hive management; and (iv) microorganism inoculation. We further investigated their effects on the pollinator behaviour and fruit production. For the artificial pollination, we identified 33 studies and categorised the techniques by pear groups and species. The artificial pollination techniques included hand pollination, hanging bottles, sprayer pollination, and UAV liquid pollination. We also added two relevant techniques widely used in preparing for artificial pollination: pollen extender selection and viable pollen detection.

Regarding the languages, we checked that the included studies had titles, abstracts, tables, and/or figure legends written in English. The whole selection process is presented in a PRISMA diagram (Fig. S5). Because some studies reported more than one pear species, we extracted the datasets at the species level for each study, regardless of cultivar, location, or experimental year. The complete datasets and reference list used for analyses are supplied in the supplementary materials.

### Data analysis

All statistical analyses and data visualization were conducted using R 4.5.1 [109]. We attempted to conduct metadata analyses for other quantitative data extracted from the reviewed studies. However, the number of studies was limited at the level of pear groups and at the taxonomic level of pollinators. For the PD and PL values, we applied the quasi-binomial model, which better fits the current case than the binomial model, for which the variance was significant [110]. In the current analysis, we did not differentiate between wild and/or managed bees due to a lack of studies on pollination reliant solely on wild insects. We fitted GLMs in the R package ‘lme4’ [111] to the PD and PL values (quasi-binomial distribution with a logit link), respectively. For each model, the fixed effect was the pear group (Asian and European pears). Specifically for the PD values in Asian pears, we applied GLMs with a fixed effect for pollination approach (insect and artificial).

For the floral morphological traits of the number of flowers per inflorescence and the flower diameter, we applied LMs in the R package ‘lme4’ [111]. For the floral rewards of pollen, we used GLMs for the number of pollen grains per anther (Poisson distribution with a log link), pollen viability (quasi-binomial distribution with a logit link), and pollen germination rate (quasi-binomial distribution with a logit link), with the fixed effect of pear group (Asian and European pears). For the floral rewards of nectar, we fitted an LM to nectar volume per flower, and a GLM to sugar concentration (quasi-binomial distribution with a logit link). For the different volatile organic compounds, we applied GLMs to the relative content of linalool, β-ocimene, methyl 2-hydroxy-3-methylpentanoate, and N-containing compounds, respectively, by using a quasi-binomial distribution with a logit link.

For the number of taxa at each taxonomic level (order, family, and genus) between Asian and European pears, we fitted GLM (Poisson distribution with a log link). The fixed effect was the pear group (Asian and European pears). For the foraging behaviours, we applied LMs to the number of visited flowers per minute, the time spent per flower, and stigmatic pollen deposition. The significance of the models was evaluated using the LRT in GLMs and the F-test in LMs. For all LMs, we examined the model assumptions of normality (using a QQ plot and the Shapiro–Wilk test) and homogeneity of variance (using Bartlett’s test). Log transformations were applied to the response variables, the number of flowers per inflorescence, and nectar volume per flower, to meet the models’ normality assumption.

*Post hoc* analysis was conducted by using estimated marginal means (EMMs) in the R package ‘multcompView’ [112] and ‘emmeans’ [113]. We used WebPlotDigitizer [114] to extract data from studies that presented results only as figures. The reviewed studies were visualised using the R packages ‘tidyverse’ [115], ‘maps’ [116], ‘packcircles’ [117], ‘ggpubr’ [118], and ‘ggtext’ [119].

## Supplementary Material

Web_Material_uhag136

## Data Availability

All data are available in the article and in the supplementary materials.
