# Supplementary material for: Bridging the gap between pollination-related floral traits and fruit production in Asian pears
Source: Hortic Res. 2026 Apr 13;13(8):uhag136. doi: 10.1093/hr/uhag136 (PMC13439133; doi:10.1093/hr/uhag136)
Supplement: Supplementary file 1 [file web_material_uhag136.zip › Supplementary material-1.docx]

**Supplementary materials**

**Bridging the gap between pollination-related floral traits and fruit production in Asian pears**

Shuxuan Jing^1^, Wufan Zhang^1^, Yibo Luo^2,*^, Jiaxing Huang^1,*^

^1^ State Key Laboratory of Resource Insects, Key Laboratory for Insect-Pollinator Biology of the Ministry of Agriculture and Rural Affairs, Institute of Apicultural Research, Chinese Academy of Agricultural Sciences, Beijing 100093, China.

^2^ State Key Laboratory of Plant Diversity and Specialty Crops, Institute of Botany, Chinese Academy of Sciences, Beijing 100093, China

^*^Co-corresponding authors:

Yibo Luo (Email: luoyb@ibcas.ac.cn)

Jiaxing Huang (Email: huangjiaxing@caas.cn)

**Running title:** Pear pollination review

**Supplementary material-1:**

**FIG. S1.** Comparison of pollinator dependence between artificial and insect pollination approaches within Asian pear group.

**FIG. S2.** Comparison of flower morphological traits between Asian and European pear groups.

**FIG. S3.** Comparison of nectar traits between Asian and European pear groups.

**FIG. S4.** Screenshot of the first page of the initial search for literature on the Web of Science.

**FIG. S5.** Prisma diagram of the selection process of pear pollination studies.

**FIG. S6.** The number of pear pollination studies investigating different techniques in relation to artificial pollination approaches in Asian pears.

**Table S1.** Pollination management practices to improve the behaviours of managed bees and fruit production.

**Reference list** of the studies included for analyses.

**Supplementary material-2:**

The datasets used in the analyses and are supplied as an Excel file:

**Table S2.** Pollinator dependence.

**Table S3.** Pollen limitation.

**Table S4.** Pollinator dependence of insect and artificial pollination in the Asian pears.

**Table S5.** Flower morphology.

**Table S6.** Pollen traits.

**Table S7.** Nectar traits.

**Table S8.** Floral volatile organic compounds.

**Table S9.** Species list for the diversity of flower visitors.

**Table S10.** Foraging behaviours.

**Table S11.** Artificial pollination techniques.

**FIG. S1.** Comparison of pollinator dependence between artificial and insect pollination approaches within Asian pear group. Error bars indicate the 95% confidence interval of the estimated marginal means (EMMs). n.s.=non-significance (*p*>0.05).

**FIG. S2.** Comparison of flower morphological traits between Asian and European pear groups: (**A**) The number of flowers per inflorescence; (**B**) Flower diameter. Error bars indicate the 95% confidence interval of the estimated marginal means (EMMs). n.s.=non-significance (*p*>0.05).

**FIG. S3.** Comparison of nectar traits between Asian and European pear groups: (**A**) Nectar volume per flower; (**B**) Nectar sugar concentration. Error bars indicate the 95% confidence interval of the estimated marginal means (EMMs). n.s.=non-significance (*p*>0.05).

**FIG. S4.** Screenshot of the first page in the initial search of literatures (N=1455) in Web of Science (accessed August 12, 2025), including the databases of CABI: CAB Abstracts^®^ (N=1417), Web of Science Core Collection (N=506), MEDLINE^®^ (N=118), Chinese Science Citation Database^SM^ (N=104), KCI-Korean Journal Database (N=24), SciELO Citation Index (N=5).

**FIG. S5.** Prisma diagram of the selection process of pear pollination studies.

**FIG. S6.** The number of pear pollination datasets investigating different techniques in relation to artificial pollination approaches in Asian pears. Pear species included *P. communis*, *P. pyrifolia*, *P. bretschneideri*, and *P. sinkiangensis*.

**Table S1.** Pollination management practices to improve the behaviours of managed bees and fruit production.

| Management practices | Improved  pollinator behaviour | Improved fruit production | Pear species | Study |
| --- | --- | --- | --- | --- |
| Bee attractant  (*e.g.*, synthetic Nasonov gland pheromone, queen mandibular pheromone, bee scent) | Abundance | Fruit size | *P. communis* | Currie *et al.*, 1992 |
|  | No effect | Fruit size | *P. communis* | Naumann *et al.*, 1994 |
|  | Abundance | No effect | *P. communis* | Neira *et al.*, 1996 |
|  | Pollen collection | Not applicable | *P. communis* | Ma *et al.*, 2015 |
|  | Abundance and visitation rate | Not applicable | *P. sinkiangensis* | Li *et al.*, 2023 |
|  |  |  |  |  |
| Floral rewards manipulation  (*e.g.*, pollen trapping, scented sucrose feeding) | Pollen collection | Not applicable | *P. bretschneideri* | Gemeda *et al.*, 2018 |
|  | Pollen collection | Fruit yield | *P. communis* | Farina *et al.*, 2022 |
|  |  |  |  |  |
| Bee hive management | Abundance | Fruit set | *P. communis* | Mayer, 1994 |
|  | Abundance and mobility | Fruit set | *P. communis* | Stern *et al.*, 2004 |
|  |  |  |  |  |
| Microorganism inoculation | Visitation rate | No effect | *P. communis* | Colda *et al.*, 2021 |

**Reference list included for different analyses (N=105)**

**Alonso-López, M.T., Garrote, P.J., and Fedriani, J.M.** (2022). Spatial isolation impacts pollinator visitation and reproductive success of a threatened self-incompatible Mediterranean tree. Acta Oecol. **117**: 103866.

**Avila, L., McCullough, C., Schiffer, A., Moreno, J., Ganjur, N., Ofenloch, Z., DuPont, T., Nottingham, L., Gerardo, N.M., and Brosi, B.J.** (2024). Effects of a field-sprayed antibiotic on bee foraging behavior and pollination in pear orchards. Agric. Ecosyst. Environ. **359**: 108757.

**Balfour, N., and Ratnieks, F.** (2017). Using the waggle dance to determine the spatial ecology of honey bees during commercial crop pollination. Agr. Forest Entomol. **19**: 210-216.

**Belien, T., Raymaekers, S., Eeraerts, M., Mommaerts, V., Claus, G., Bogen, C., Piot, N., Smagghe, G., Spanoghe, P., and Bylemans, D.** (2021). Towards integrated pest and pollinator management in intensive pear cultivation: A case study from Belgium. Insects **12**: 901.

**Branzanti, E.C.** (1964). Observations on the pollination of the pear variety Abate Fetel. Rivista della Ortoflorofrutticoltura Italiana **48**: 424-427.

**Callan, N.W., and Lombard, P.B.** (1978). Pollination effects on fruit and seed development in “Comice” pear. J. Am. Soc. Hortic. Sci. **103**: 496-500.

**Choudhary, A., Singh, J., Kaur, R., and Chhuneja, P.K.** (2021). Augmenting pear yield through *Apis mellifera* pollination. Indian J. Entomol. **83**: 43-47.

**Colda, A., Bossaert, S., Verreth, C., Vanhoutte, B., Honnay, O., Keulemans, W., and Lievens, B.** (2021). Inoculation of pear flowers with Metschnikowia reukaufii and Acinetobacter nectaris enhances attraction of honeybees and hoverflies, but does not increase fruit and seed set. PLoS ONE **16**: e0250203.

**Currie, R.W., Winston, M.L., Slessor, K.N., and Mayer, D.F.** (1992). Effect of synthetic queen mandibular pheromone sprays on pollination of fruit crops by honey bees (Hymenoptera: Apidae). J. Econ. Entomol. **85**: 1293-1299.

**Dahliwal, G.S., Aulakh, P.S., and Jawanda, J.S.** (1982). Investigations of floral biology in pear (*Pyrus communis* L.). II. Studies on pollen germination and pollination in relation to fruit-set. Punjab Horticultural Journal **22**: 164-168.

**de Castro, B., Marodin, G.A.B., and Ferreira, P.H.G.** (2021). Floral visitors of European pear trees Clapp’s Favourite in southern Brazil. In: I international symposium on reproductive biology of fruit tree species. Acta Hortic. **1342**, pp. 221-228.

**Dolan, B.J., Kalkstein, A.L., Haase, M., Chernisky, P., Niese, A., Hicks, A.N., and Miller, D.** (2022). Pollinator communities of planted and feral *Pyrus calleryana*. Ecologies **4**: 1-10.

**Endo, K., Hiraguri, T., Kimura, T., Shimizu, H., Shimada, T., Shibasaki, A., Suzuki, C., Fujinuma, R., and Takemura, Y.** (2024). Estimation of the amount of pear pollen based on flowering stage detection using deep learning. Sci. Rep. **14**: 13163.

**Faoro, I.D., and Orth, A.I.** (2010). Fruit set influenced by the occurrence of parthenocarpy in Asiatic pear tree cultivars in two locations in Santa Catarina State, South Brazil. VIII International Symposium on Temperate Zone Fruits in the Tropics and Subtropics, Florianopolis, Brazil. Acta Hortic. **872**, pp. 261-266.

**Farina, W.M., Arenas, A., Díaz, P.C., Susic Martin, C., and Corriale, M.J.** (2022). In-hive learning of specific mimic odours as a tool to enhance honey bee foraging and pollination activities in pear and apple crops. Sci. Rep. **12**: 20510.

**Farkas, Á., and Orosz-Kovács, Z.** (2004). Primary and Secondary Attractants of Flowers in Pear Pyrus betulifolia. In: Proceeding XXVI IHC-deciduous Fruit and nut trees. Webster AD, ed. Acta Hortic. **636**: 317-324.

**Farkas Á, Orosz-Kovács Z., Szabó, L.G., and Bubán, T.** (2000). Floral attractivity of pear cultivar ‘Cinderi’. Int. J. Hortic. Sci. **6**: 102-109.

**Fiala, J., Zezulová, E., and Nečas, T.** (2024). Evaluation of the genome size and ploidy level of pears (*Pyrus* spp.) in relation to their morphological traits. Horticulturae **10**: 1241.

**Fountain, M.T., Mateos-Fierro, Z., Shaw, B., Brain, P., and Delgado, A. (2019).** Insect pollinators of conference pear (*Pyrus communis* L.) and their contribution to fruit quality. J. Pollinat. Ecol. **25**: 103-114.

**Gemeda, T.K., Shao, Y., Wu, W., Yang, H., Huang, J., and Wu, J. (2017).** Native honey bees outperform adventive honey bees in increasing Pyrus bretschneideri (Rosales: Rosaceae) pollination. J. Econ. Entomol. **110:** 2290-2294.

**Gemeda, T.K.** (2018). *Pollination application of honey bees in pear orchard*. PhD Thesis, Chinese Academy of Agricultural Sciences, China.

**Giejdasz, K., Fliszkiewicz, M., Dylewski, Ł., and Banaszak‐Cibicka, W.** (2025). Optimizing pear orchard productivity through managed *Osmia bicornis* L.: A study on pollination efficiency and its role in geitonogamy. Ann. Appl. Biol. aab.70030.

**Griggs, W.H., and Vansell, G.H.** (1949). The use of bee-collected pollen in artificial pollination of deciduous fruits. Proceedings American Society for Horticultural Science **54**: 118-124.

**Haragsim, O.** (1974). Nectar production of pear trees and the activity of bees in pollinating their flowers. Vedecke Prace Vyzkumneho Ustavu Vcelarskeho v Dole u Libcic **6**: 9-23.

**Holland, J.M., Sutter, L., Albrecht, M., Jeanneret, P., Pfister, S.C., Schirmel, J., Entling, M.H., Kaasik, R., Kovacs, G., Veromann, E., Bartual, A.M., Marini, S., Moonen, A-C., Szalai, M., Helsen, H., Winkler, K., Lof, M.E., Van Der Werf, W., McHugh, N.M., Smith, B.M., Wallis, D.W., and Cresswell, J.E.** (2020). Moderate pollination limitation in some entomophilous crops of Europe. Agric. Ecosyst. Environ. **302**: 107002.

**Hooper, C.H.** (1919). Notes on insect visitors to fruit blossoms. Journal of Pomology and Horticultural Science **1**: 116-124.

**Huang, X., Zhang, Q., Sheikh, U., Wang, Y., and Zheng, L.** (2024). Bumblebee foraging dynamics and pollination outcomes for cherry tomato and pear varieties in Northern China. Insects **15**: 216.

**Hünicken, P.L., Morales, C.L., García, N., and Garibaldi, L.A.** (2020). Insect pollination, more than plant nutrition, determines yield quantity and quality in apple and pear. Neotrop. Entomol. **49**: 525-532.

**Hutson, R.** (1925). The honey bee as an agent in the pollination of pears, apples and cranberries. J Econ. Entomol. **18**: 387-391.

**Jacquemart, A.-L., Michotte-Van Der Aa, A., and RaspÉ O.** (2006). Compatibility and pollinator efficiency tests on *Pyrus communis* L. cv. ‘Conference.’ J. Hortic. Sci. Biotechnol. **81**: 827-830.

**Jafarzadeh, S., and Iskandar, E.** (2024). Comparative study of pollen morphology and fertility in *Pyrus* L. species under in situ and ex situ conditions in Greater Caucasus, Azerbaijan. Y Alibek, ed. BIO Web of Conferences **100**: 03006.

**Ji, R., Wang, L., Wang, Z., Gou, C., Hao, H., and Feng, H.** (2023). Effects of chitosan to pollination solution on fruit setting rate and fruit quality of Korla Fragrant Pear. Xinjiang Agricultural Sciences **60**: 1208-1215.

**Jiang, W., Han, H., Wong, L., and Huang, W.** (2004). Study on pollination and bearing habit of 16 new pear cultivars. South China Fruits **33**: 42-43.

**Jiang, Y., Wei, J., and Zhang, Q.** (2015). Comparative studies on pollens characters and floral organ structures of different cultivars and strains of pear. Journal of Henan Agricultural Sciences **44**: 100-107 (in Chinese).

**Kim, J.K., Lee, H.C., Yoon, I.K., and Moon, B.W.** (2003). A newly-developed pollen extender used for artificial pollination in fruit trees. Korean J. Hortic. Sci. Technol. **21**: 329-332.

**La Franchie, E.J.** (2002). Artificial pollen application on nashi cultivar ‘Housui’. In: Proceedings of the international symposium on Asian pears commemorating the 100^th^ anniversary of Nijisseiki pear, vols I and II. Iwahori S, Gemma H, Tanabe K, Webster AD, White AG, eds. Acta Hortic. **587**: 599-604.

**Langridge, D.F., and Jenkins, P.T.** (1975). A study on pollination of Winter Nelis pears. Aust. J. Exp. Agric. Anim. Husb. **15**: 105-107.

**Langridge, D.F., Jenkins, P.T., Whan, J.H., and Goodman, R.D.** (1976). Further studies on pollination of Packham’s Triumph pears. J. Apic. Res. **15**: 155-160.

**Lee, K.Y., Yim, S.H., Seo, H.J., Kim, S.Y., and Yoon, H.J.** (2016). The influence of insect pollination and artificial pollination on fruit quality and economic profit in the ‘Niitaka’ pear (*Pyrus pyrifolia* Nakai). Korean J. Org. Agric. **24**: 759-771.

**Lee, K.Y., Yim, S.H., Seo, H.J., Shin, M., and Yoon, H.J.** (2017). Pollination efficiency of honeybees (*Apis mellifera* L.) and bumblebees (*Bombus terrestris* L.) in different cultivars of Asian pear (*Pyrus pyrifolia* Nakai). Journal of Apiculture **32**: 303-314.

**Lewis, T., and Smith, B.D.** (1969). The insect faunas of pear and apple orchards and the effect of windbreaks on their distribution. Ann. Appl. Biol. **64**: 11-20.

**Li, L., Gao, L., Xu, J., Ji, M., Liu, H., Zeng, Q., and Hao, B.** (2025). Breeding report of a new mid-ripening red pear cultivar Canyu. Journal of Fruit Science **42**: 668-671 (in Chinese).

**Li, Q., Bianchi, F.J.J.A., Lu, Y., Liu, B., and van der Werf, W.** (2019). Pollination and biological control services in Korla pear (*Pyrus bretschneideri* Rehd). IOBC/WPRS Bulletin **143**: 79-83.

**Li, Q., Liu, B., Van Der Werf, W., Bianchi, F.J.J.A., and Lu, Y.** (2025). Species-specific responses to landscape composition of wild bees in pear orchards. Entomologia **45**: 753-763.

**Li, Q., Sun, M., Liu, Y., Liu, B., van der Werf, W., Bianchi, F.J.J.A., and Lu, Y.** (2023). Synthetic Nasonov gland pheromone enhances abundance and visitation of honeybee, Apis mellifera, in Korla fragrant pear, Pyrus sinkiangensis. Agric. For. Entomol. **25**: 365-374.

**Li, Q., Sun, M., Liu, Y., Liu, B., Bianchi, F.J.J.A., van der Werf, W., and Lu, Y.** (2022). High pollination deficit and strong dependence on honeybees in pollination of Korla Fragrant Pear, *Pyrus sinkiangensis*. Plants **11**: 1734.

**Liu, L., Liu, Z., Han, H., Jiang, Y., He, X., Liu, Y., Wang, D., Guo, X., and Liang, Z.** (2023). Influence of different liquid spray pollination parameters on pollen activity of fruit trees-pear liquid spray pollination as an example. Horticulturae **9**: 350.

**Lukas, K., Harig, T., Schulz, S., Hadersdorfer, J., and Dötterl, S.** (2019). Flowers of European pear release common and uncommon volatiles that can be detected by honey bee pollinators. Chemoecology **29**: 211-223.

**Ma, W., Long, D., Wang, Y., Li, X., Huang, J., Shen, J., Su, W., Jiang, Y., and Li, J.** (2021). Electrophysiological and behavioral responses of Asian and European honeybees to pear flower volatiles. J. Asia-Pac. Entomol. **24**: 221-228.

**Ma, W.H., Shao, Y.Q., Zhao, H.T., Tian, S.H., Meng, J., Yang, S.S., Du, Y.L., and Jiang, Y.S.** (2015). Using bee attractants to improve honeybee foraging on Dangshan pear (Pyrus communis L.). J. Agric. Sci. **17**: 1551-1558.

**Maccagnani, B., Ladurner, E., Santi, F., and Burgio, G.** (2003). *Osmia cornuta* (Hymenoptera, Megachilidae) as a pollinator of pear (*Pyrus communis*): fruit- and seed-set. Apidologie **34**: 207-216.

**Makimura, F., Tsuruta, M., Yamasaki, K., and Mukai, Y.** (2015). Visitors to flowers of the endangered Callery pear, *Pyrus calleryana* Decne. var. dimorphopheylla (Makino) Koidz., in the Tado population, Mie Prefecture. Japanese Journal of Conservation Ecology **20**: 197-202.

**Mayer, D.F.** (1994). Sequential introduction of honey bee colonies for pear pollination., in: Sugar D, ed., Acta Hortic. pp. 267-269.

**Mayer, D.F., and Lunden, J.D.** (1997). A comparison of commercially managed bumblebees and honey bees (Hymenoptera: Apidae) for pollination of pears. Acta Hortic. **437**: 283-288.

**Miliczky, E.R., Mayer, D.F., and Lunden, J.D.** (1990). Notes on the nesting biology of *Andrena (Melandrena) nivalis* Smith (Hymenoptera: Andrenidae). J. Kans. Entomol. Soc. **63**: 166-174.

**Monzón, V.H., Bosch, J., and Retana, J.** (2004). Foraging behavior and pollinating effectiveness of *Osmia cornuta* (Hymenoptera: Megachilidae) and *Apis mellifera* (Hymenoptera: Apidae) on “Comice” pear. Apidologie **35**: 575-585.

**Mori, S., Mitsuhata, M., and Yokoi, T.** (2024). Protein/Lipid ratio of pollen biases the visitation of bumblebees (*Bombus ignitus* Smith) to male-fertile cultivars of the Japanese pear (*Pyrus pyrifolia* Nakai). PLoS ONE 19: e0297298.

**Murakami, S., Hashimoto, N., Araki, Y., and Yamane, S.** (2020). Artificial pollination of Japanese pear and kiwifruit using electrostatic pollen dusting machines. Horticultural Research (Japan) **19**: 365-372.

**Nakamura, S., Taki, H., Arai, T., Funayama, K., Furihata, S., Furui, Y., Ikeda, T., Inoue, H., Kagawa, K., Kishimoto, H., Kohyama, M., Komatsu, M., Konuma, A., Nakada, K., Nakamura Suguru Sawamura, N., Sonoda, S., Sueyoshi, M., Toda, S., Yaginuma, K., Yamamoto, S., Yoshida, K., Yokoi, T., and Toyama, M.** (2023). Diversity and composition of flower-visiting insects and related factors in three fruit tree species. Biodiversity Data Journal **11**: e100955.

**Naumann, K., Winston, M.L., Slessor, K.N., and Smirle, M.J.** (1994). Synthetic honey bee (Hymenoptera: Apidae) queen mandibular gland pheromone applications affect pear and sweet cherry pollination. J. Econ. Entomol. **87**: 1595-1599.

**Neira, C.M., Nallar, F.J., Pessot, Z.R., and Carrillo, L.R.** (1996). Pollinating activity of Apis mellifera L., in pear orchards, under the influence of two attractants. Agro. Sur. **24**: 167-176 (in Spanish).

**Niu, Y., Chen, X., Zhou, W., Li, W., Zhao, S., Nasir, M., Dong, S., Zhang, S., and Liao, K.** (2019). Genetic relationship between the ‘Korla fragrant pear’ and local pear varieties in xinjiang based on floral organ characteristics. Sci. Hortic. **257**: 108621.

**Ohno, M., Takahashi, E., and Teranuma. T. (1964).** Studies on the efficiency of artificial pollination in fruit growing. Utilization of pollen-stimulating substances in artificial pollination. Journal of Japanese Society for Horticultural Science **33**: 1-7.

**Okada, I., Niijima, K., and Ashizawa, I. (1982).** Pear fruit set obtained by using pollen loads from honeybees. Honeybee Science **3**: 75-76.

**Okamoto, K., Notoyama, A., Muramatsu, Y., Kagawa, K., Mikawa, Y., Aizawa, M., Sueyoshi, M., Mita, T., Toyama, M., and Sonoda, S.** (2024). Pollination efficiency of *Apis mellifera* (Hymenoptera: Apidae), solitary bees (Hymenoptera), and flies (Diptera) in an insect-pollinated Japanese pear orchard. J. Asia-Pac. Entomol. **27**: 102242.

**Panov, V., and Petkov, V.** (1975). The effectiveness of the pollination of pears by bees. Gradinarska I Lozarska Nauka **12**: 33-40.

**Paul, A., Kaur, N., and Gill, P.P.S.** (2023). Reproductive phenology of four pear cultivars grown under sub-tropics of Punjab. Plant Physiol. Rep. **28**: 556-567.

**Péter, J.** (1972). Körte. (Pear.) In: Halmágyi L., Keresztesi B. (szerk.) A méhlegelő (The bee pasture) Académiai Kiadó, Budapest, p. 471-474.

**Quinet, M., and Jacquemart, A.-L.** (2017). Cultivar placement affects pollination efficiency and fruit production in European pear (*Pyrus communis*) orchards. Eur. J. Agron. **91**: 84-92.

**Quinet, M., Warzée, M., Vanderplanck, M., Michez, D., Lognay, G., and Jacquemart A-L.** (2016). Do floral resources influence pollination rates and subsequent fruit set in pear (*Pyrus communis* L.) and apple (*Malus* x *domestica* Borkh) cultivars? Eur. J. Agron. **77**: 59-69.

**Quinet, M., and Jacquemart, A.-L.** (2020). Troubles in pear pollination: Effects of collection and storage method on pollen viability and fruit production. Acta Oecol. **105**: 103558.

**Ren, R., Sun, H., Zhang, S., Zhao, H., Wang, L., Su, M., and Sun, T.** (2024). FPG-YOLO: A detection method for pollenable stamen in “Yuluxiang” pear under non-structural environments. Sci. Hortic. **328**: 112941.

**Sakamoto, D., Hayama, H., Ito, A., Kashimura, Y., Moriguchi, T., and Nakamura, Y.** (2009). Spray pollination as a labor-saving pollination system in Japanese pear (*Pyrus pyrifolia* (Burm.f.) Nakai): Development of the suspension medium. Sci. Hortic. **119**: 280–285.

**Sakamoto, D., Kusaba, S., and Nakamura, Y.** (2016). Addition of Forchlorfenuron to spray pollination media increases fruit set in ‘Hosui’ Japanese pear. HortTechnology **26**: 185-190.

**Seo, H.-J., Song, J., Yoon, H.J., and Lee, K.Y.** (2019). Effects of nectar contents on the foraging activity of honeybee (*Apis mellifera*) on Asian pear (*Pyrus pyrifolia* Nakai). Sci. Hortic. 245: 185-192.

**Seo, H. J., Park, Y., Choi, E. D., Lee, B., Hong, S. S., Sawant, S. S., Yu, S.H., and Lee, C. G.** (2025). Effects of drone-assisted artificial pollination on fruit set and characteristics in Asian pear. J. Horti. Sci. Biotech. 1-10.

**Sharma, P., Coyle, D.R., Kunkel, D., Palmer, J.F., Campbell, J.W., Timilsina, N., and Hartshorn, J.** (2025). Pollinator Community Composition and Diversity in Callery Pear Stands in South Carolina. Southeastern Naturalist 24.

**Shen, H.** (2023). Study of *Apis cerana cerana* and *Apis mellifera ligustica* responses to the volatile compounds of different flowers. Master Thesis (in Chinese). Shanxi Agricultural University.

**Shen, G., Wang, X., Luo, J., Zhang, S., Qian, P., and Jin, F.** (2008). Effects of greenhouse culture on pear pollen quantity per anther and pollen viability. Acta Agriculturae Shanghai **24**: 54-57 (in Chinese).

**Simidchiev, T.** (1978). Full utilization of honeybees. Ovoshcharstvo **57**: 9-11.

**Smessaert, J., Honnay, O., and Keulemans, W.** (2019). Monitoring pollinator activity in an apple and pear orchard, linked with the analysis of the nectar composition. In: Ortega Pastor, E., Halasz, J., De Franceschi, P., Eds. Proc. II Int. Workshop on Floral Biology and S-Incompatibility in Fruit Species. Acta Hortic. 1231, pp. 59-65.

**Sonoda, S., Kagawa, K., Furui, Y., Nakada, K., Koyama, M., Toda, S., Sugiura, N., Nakamura, S., Sueyoshi, M., Mita, T., and Toyama, M.** (2022). Mutual complementarity among diverse pollinators as a mechanism underlying open insect pollination in Japanese pear orchards. J. Appl. Entomol. **146**: 498-510.

**Stern, R.A., Goldway, M., Zisovich, A.H., Shafir, S., and Dag, A.** (2004). Sequential introduction of honeybee colonies increases cross-pollination, fruit-set and yield of ‘Spadona’ pear (Pyrus communis L.). J. Hortic. Sci. Biotechnol. **79**: 652-658.

**Su, W., Ma, W., Zhang, Q., Hu, X., Ding, G., Jiang, Y., and Huang, J.** (2022). Honey bee foraging decisions influenced by pear volatiles. Agriculture **12**: 1074.

**Sun, W., Chen, C., Liu, T., Liang, H., Tian, L., Fu, X., Niu, M., Huang, S., and Hu, F.** (2024). YOLOv8-pearpollen: Method for the lightweight identification of pollen germination vigor in pear trees. Agriculture **14**: 1348.

**Teng, M., Chen, L., Liu, L., Qi, K., Zhang, S., and Wu, J.** (2015). Investigation of flowering date and fruit setting trait in different pear cultivars in Nanjing region. Journal of Fruit Science **32**: 572-582 (in Chinese).

**Tepedino, V.J., Alston, D.G., Bradley, B.A., Toler, T.R., and Griswold, T.L.** (2007). Orchard pollination in Capitol Reef National Park, Utah, USA. Honey bees or native bees? Biodivers. Conserv. **16**: 3083-3094.

**van den Eijnde J.** (1991). Research on beefsteak tomatoes. Let bumble-bees carry on undisturbed. Groenten + Fruit, Glasgroenten **1**: 13-15.

**Van den Eijnde, J., Steen, J., and van der, and Smeekens, C.** (1994). Bumble bees contribute little to pear pollination. Fruitteelt (Den Haag) 84: 30-31.

**Vaziritabar, S., Oshidari, S., and Esmaeilzade, S.M.** (2015). Comparative foraging behavior of eastern honeybee, (*Apis cerana* F) and western honeybee, (*Apis mellifera carnica*) in pollinating pear and apricot flowers in Taleghan, Iran. Journal of Biodiversity and Environmental Sciences 7: 136-149.

**Wang, J, and Wang, O.** (2002). Artificial pollination techniques for pear. South China Fruits **31**: 40-42 (in Chinese).

**Wang, J., Wang, Y., Wang, H., Li, X., Wang, Y., Wang, Y., and Li, Y.** (2023). Pollination effects of pear trees by honeybee and its influencing factors. Asian Agricultural Research **15**: 59-62.

**Wang, R., Cui, H., Guo, T., He, T., Zhang, B., Cheng, J., and Mansur, N.** (2022). Comparative study on biological characteristics, shoot growth characteristics and fruit quality characteristics of different pear varieties during flowering period. Xinjiang Agricultural Sciences, **59**: 2652-2660 (in Chinese).

**Wang, T., Zhao, Y., Li Pang, L.L., and Cheng, Q.** (2024). Evaluation method and design of greenhouse pear pollination drones based on grounded theory and integrated theory. PLoS ONE 19, e0311297.

**Wang, Y., Bai, R., Lu, X., Quan, S., Liu, Y., Lin, C., Wei, J., Su, Y., and Yao, R*.*** (2022). Pollination parameter optimization and field verification of UAV-based pollination of ‘Kuerle Xiangli.’ Agronomy **12**: 2561.

**Wei, J., Jiang, Y., and Xie, H. (2024).** Optimization of flight operation parameters for supplementary pollination in Kuerlexiangli pear using an unmanned aerial vehicle. Journal of Fruit Science **41**: 162-173 (in Chinese).

**Westwood, M.N., Stephen, W.P., and Cordy, C.B.** (1966). The possibility of wind pollination in pear. Hort. Sci. **1**:28-29.

**Whiting, M., Taylor, K., and Das, P.** (2022). Supplemental artificial pollination can improve fruit set in tree fruit, in: Musacchi, S. (Ed.), Presented at the XII international symposium on integrating canopy, rootstock and environmental physiology in orchard systems, pp. 121-127.

**Wilson, G.F.** (1929). Pollination of hardy fruits: insect visitors to fruit blossoms. Ann. Appl. Biol. **16**: 602-629.

**Xu, J., Wuyun, T., and Wang, G.** (2015). Study on genetic diversity of wild Pyrus ussuriensis flower sharp in China. Journal of Central South University of Forestry & Technology **35**: 27-30, 36 (in Chinese).

**Xu, Y., Zhang, X., Zhuang, H., Dong, W., and Gong, P.** (2022). Determination of Dangshan pear pollen viability using near-infrared spectroscopy. Xinjiang Agricultural Sciences **59**: 617-624 (in Chinese).

**Yang, J., Yang, H., Xie, W., Hao, H., Feng, H., and Wang, L.** (2021). Parameter optimization and economic analysis of unmanned aerial vehicle (UAV) assisted liquid pollination for Kuerlexiangli pear. Journal of Fruit Science **38**: 1691-1698 (in Chinese).

**Zhang, J.-C., Liu, Q., Huang, J.-X., Gao, F., Chen, M., Ma, W.-H., and Jiang, Y.-S.** (2022). Effects of flower characteristics of pear and rape on flower-visiting preference of honeybees. Journal of Environmental Entomology **44**: 910-920 (in Chinese).

**Zhang, X., Wang, F., Cao, Q., Wu, M., Guo, B., and Guo, Y.** (2025). Analysis of physiological characteristics of early fertilization and pollination effects of pear (*Pyrus bretschneideri* cv. Dangshansu) under different pollination methods. Journal of Shanxi Agricultural Sciences **53**: 119–128 (in Chinese).

**Zhao, B.-M., Zhao, J., Tang, Z.-L., and Zhang, Z.** (2014). Pollination technique of hanging bottles for Cuiguan pear. Journal of Shanghai Jiaotong University – Agricultural Science **32**: 36-41 (in Chinese).
